# Non-oral dopaminergic therapies for Parkinson’s disease: current treatments and the future

**DOI:** 10.1038/npjparkd.2016.23

**Published:** 2016-12-01

**Authors:** K Ray Chaudhuri, Mubasher A Qamar, Thadshani Rajah, Philipp Loehrer, Anna Sauerbier, Per Odin, Peter Jenner

**Affiliations:** 1 National Parkinson Foundation International Centre of Excellence, Department of Neurology, King’s College Hospital, London, UK; 2 Department of Basic and Clinical Neuroscience, The Maurice Wohl Clinical Neuroscience Institute, King’s College, London, UK; 3 Department of Neurology, University Hospital Cologne, Cologne, Germany; 4 Department of Neurology, Lund University, Skane University Hospital, Lund, Sweden; 5 Klinikum-Bremerhaven, Bremerhaven, Germany; 6 Neurodegenerative Diseases Research Group, Institute of Pharmaceutical Sciences, Faculty of Life Sciences and Medicine, King’s College London, London, UK

## Abstract

Dysfunction of the gastrointestinal tract has now been recognized to affect all stages of Parkinson’s disease (PD). The consequences lead to problems with absorption of oral medication, erratic treatment response, as well as silent aspiration, which is one of the key risk factors in developing pneumonia. The issue is further complicated by other gut abnormalities, such as small intestinal bacterial overgrowth (SIBO) and an altered gut microbiota, which occur in PD with variable frequency. Clinically, these gastrointestinal abnormalities might be associated with symptoms such as nausea, early-morning “off”, and frequent motor and non-motor fluctuations. Therefore, non-oral therapies that avoid the gastrointestinal system seem a rational option to overcome the problems of oral therapies in PD. Hence, several non-oral strategies have now been actively investigated and developed. The transdermal rotigotine patch, infusion therapies with apomorphine, intrajejunal levodopa, and the apomorphine pen strategy are currently in clinical use with a few others in development. In this review, we discuss and summarize the most recent developments in this field with a focus on non-oral dopaminergic strategies (excluding surgical interventions such as deep brain stimulation) in development or to be licensed for management of PD.

## Introduction

**Figure 1:**
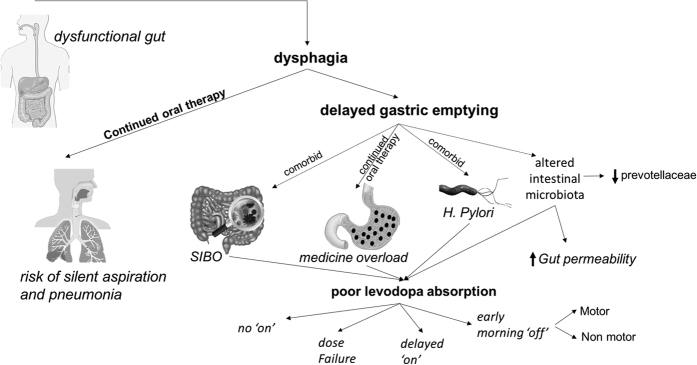
The problems of oral therapy in Parkinson’s disease in relation to various pathologies within the upper gastrointestinal system. Poor levodopa absorption could be the chief cause of many variants of levodopa-induced motor fluctuations. *H. Pylori, Helicobacter pylori*; SIBO, small intestine bacterial overgrowth.

Dysfunction of the gut at all levels is now recognized in Parkinson’s disease (PD) from pre-clinical, early and advanced stages.^1–6^ In particular, dysphagia may lead to silent aspiration and delayed gastric emptying, which are problems where the latter has been predominantly implicated in the pathogenesis of motor complications, such as delayed “on” and non “on” responses to oral dopaminergic therapy in PD.^3,7^ In addition, silent aspiration is a risk factor for pneumonia in PD.^8^ Furthermore, the role of other gut abnormalities such as small intestinal bacterial overgrowth (SIBO)^9^ as well as altered gut microbiota^10^ and interference with oral drug therapy in PD is unclear. A further manifestation of problems with gastric absorption of oral therapy is the increasing recognition of early-morning “off” periods in PD, which can only be effectively managed by non-oral therapies^11,12^ (Figure 1).

Non-oral therapies have, therefore, been actively investigated and developed over the past decade, key successes being the transdermal rotigotine (RTG) patch, infusional therapies with apomorphine, intrajejunal levodopa, and the apomorphine pen strategy, all of which are currently in clinical use. With the increasing recognition of the extent of multilevel gastrointestinal dysfunction in PD, several future dopamine replacement therapy-based strategies have focussed on novel non-oral strategies for management of motor and non-motor aspects of PD (Table 1). In this review, we summarize the recent developments in this field focussing on non-oral dopamine replacement therapy strategies in development or to be licensed for management of PD.

## Levodopa-based treatment strategies

### Intrajejunal Levodopa infusion (Duodopa/Duopa (USA))

This therapy is now in widespread clinical use and a detailed review is beyond the scope of this article. Levodopa-carbidopa (LD-CD) intrajejunal infusion is an intestinal gel that is administered continuously into the primary site of levodopa absorption, the proximal jejunum. This is achieved via a percutaneous endoscopic gastrojejunostomy tube connected to a portable infusion pump. Duodopa was first launched in Sweden in 2004, after pioneering work by Professor Aquilonius and colleagues in Upsaala university, and it has now been on the market for 11 years.^13^

Recently, a double-blind, double-dummy, active-controlled, parallel group, multicentre study that evaluated the efficacy, safety, and tolerability of Duodopa against LD-CD 100/25 mg tablets. They reported that Duodopa significantly reduced “off” periods and increased “on” time without troublesome dyskinesias.^14^ Tolerability of Duodopa has also been shown in a phase 3, 12-month, open-label, single-arm, multicentre trial by Fernandez and colleagues^15^ showing a good tolerability at 54 weeks.^15^ Early intervention is associated with procedural complications such as pain, local site infection, and tube detachment, whereas in the long-term problems such as weight loss, vitamin B12 deficiency, and polyneuropathy have been reported.^16^ Non-motor outcomes after Duodopa have also been reported in open label,^17^ as well as comparative and registry-based studies.^18–20^ In addition, a study by Zibetti and colleagues^21^ suggest that Duodopa appears to have a sustained beneficial effect on sleep (excessive daytime sleepiness), fatigue, urinary function, and pain.^21^ Low rates of impulse control disorder (ICD) have been reported with Duodopa, and some would consider Duodopa specifically in cases with troublesome ICD.^18,20^

### Intrajejunal TriGel infusion

TriGel is a new product that is composed of the LD-CD intestinal gel with an additional ingredient of entacapone.^22^ In tablet form, entacapone, a well-established catechol-O-methyl transferase (COMT) enzyme inhibitor, has shown to increase the bioavailability of levodopa by extending its half-life.^22^ Entacapone is commonly used in clinical practice and, by adding entacapone to the LD-CD intestinal gel, the sponsors are aiming to explore whether TriGel has an advantage over LD-CD intrajejunal infusion in terms of longer benefit per day and potentially reducing the cost.

### Inhaled Levodopa (CVT-301)

CVT-301 is a newly designed drug for self-administered levodopa inhalation therapy. The dry powder aerosol contains levodopa to treat predictable motor and refractory “off” periods in PD.^23^ Pulmonary absorption gives instant presentation of levodopa to the absorptive membrane, which has a large surface area and low metabolic activity,^24^ hence avoiding the variability in gastrointestinal absorption, poor pharmacokinetics, and consequent delayed “on”, or no “on”, or even dose failures with oral levodopa therapy. A Phase 3 trial began enrolling in late 2014 and the sponsor company intends to file for a New Drug Application with the US Food and Drug Administration in early 2017.^25,26^ Studies had demonstrated a rapid elevation of levodopa plasma levels along with a statistically significant reduction in the Unified PD rating scale (UPDRS) III motor score after 10 minutes (min) min for up to 60 min relative to placebo.^23,27^ The inhalation therapy of CVT-301 was well tolerated, whereby no increase in dyskinesia in comparison with placebo could be detected in a Phase 2b trial.^25^ The most common side effects seen were dizziness, headaches, and coughs. Importantly, thus far, no negative implications on cardiovascular or lung functions have been reported, although the long-term effects of chronic levodopa exposure to pulmonary mucosa and development of dyskinesia are unknown.^27^

**Figure 2:**
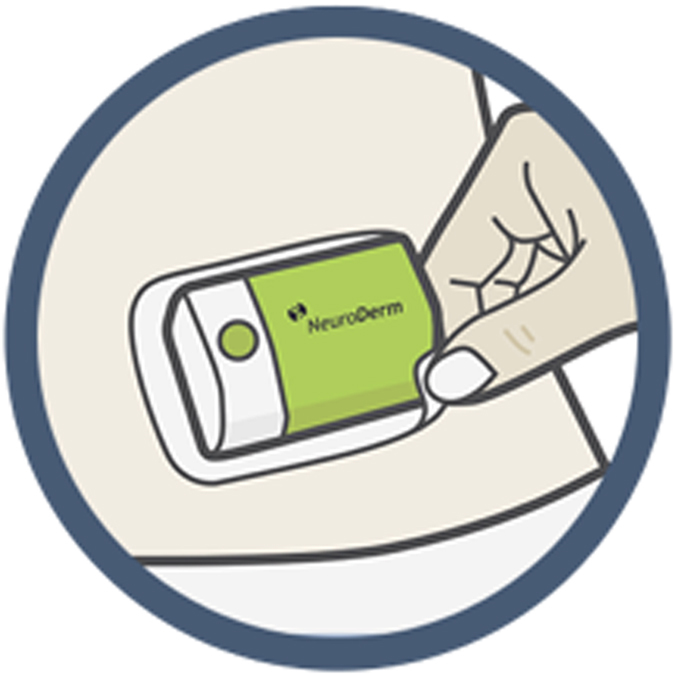
Levodopa patch–pump^29^ (permission granted by NeuroDerm).

**Figure 3:**
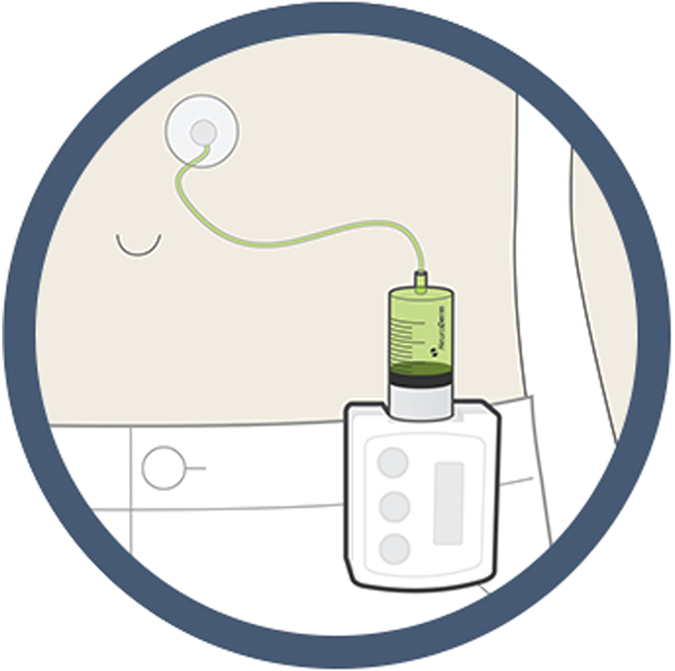
Levodopa belt pump^29^ (permission granted by NeuroDerm).

ND0612 is a novel liquid formulation of LD-CD, which has been developed for subcutaneous delivery for the treatment of moderate (ND0612L) and severe (ND0612H) PD. The subcutaneous route administration of ND0612 shows sustained levodopa plasma levels, thus offering continuous drug delivery.^28^ Subcutaneous delivery is achieved either via a novel belt pump in ND0612L or ND0612H, whereby a patch–pump (pump is attached to a transdermal patch and operated by a pump) system is in development for ND0612L (Figures 2 and 3).^29^ Currently, three different studies involving ND0612 are recruiting participants and patients. First, a multicenter, open-label Phase 2 trial assesses the long-term safety (12 months) of ND0612H in advanced PD (Hoehn and Yahr scale ⩽3).^30^ Second, another Phase 2 study investigates efficacy, pharmacokinetics, safety, and tolerability of two dosing regiments of ND0612H in a multicenter, parallel-group, rater-blinded, and randomized manner.^30^ Third, an open-label Phase 1 trial compares bioavailability of levodopa between subcutaneous delivery of ND0612 and nasojejunal-infused LD-CD intestinal gel, and seeks to identify optimal concentration of carbidopa.^31^ Previous clinical trials (including two Phase 1 and one Phase 2a study) could demonstrate that continuous subcutaneous ND0612 delivery yielded steady-state plasma concentrations estimated to be in a therapeutic window.^32^ Furthermore, a Phase 2 trial with N0612L showed reduced “off” time in clinic of 2.42±2.62 h (mean±s.d.) compared with placebo, and was accompanied by a small decrease in troublesome dyskinesia. Improvements of quality of sleep and life, measured by Panic Disorder Severity Scale and the PD Questionnaire scores, were detected.^28^ Safety analysis within the same study showed good tolerability and revealed transient local skin reactions as main adverse events. No systemic adverse reactions, in particular dyskinesia or psychiatric symptoms, were reported^28^

## Non-Levodopa-based therapies

### Transdermal rotigotine-patch

Transdermal RTG patch has been in clinical use since the early 2000 for adjunctive and initiating therapy for PD. RTG is a non-ergot dopamine agonist with its activity spanning D1 through D5 receptors, in addition to adrenergic and serotonergic sites. The transdermal approach provides a continuous delivery of RTG with stable and steady plasma levels over 24 h with a single daily application and its doses ranging from 2 to 16 mg/day.^33^ The motor efficacy, safety, and tolerability of RTG therapy have been demonstrated in several 6-month studies involving early and advanced PD patients.^34–37^ RTG patch was one of the first products to be tested for non-motor efficacy, and the RECOVER study confirmed the beneficial effects of RTG patch on night-time symptoms of PD as measured by the PD sleep scale.^38^ Other key beneficial effects of RTG patch on non-motor symptoms include dopamine-fluctuation-related pain as well as mood and anhedonia.^39,40^ A lower rate of ICD has been described with RTG patch compared with other conventional oral dopamine agonists.^41^ Skin reactions may complicate therapy, while in some, neuropsychiatric complications may occur.

### Subcutaneous apomorphine injection and infusion

**Figure 4:**
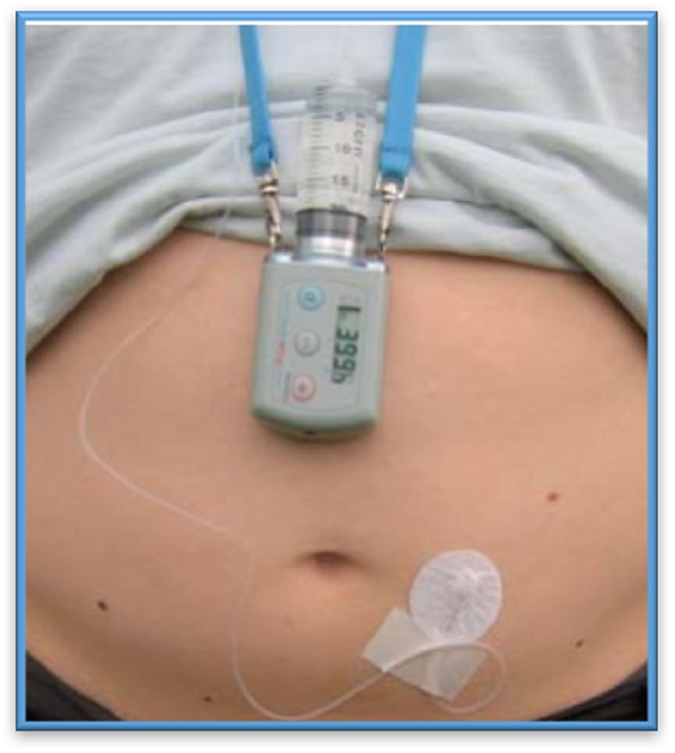
Apomorphine subcutaneous infusion.

Modern clinical experience of using apomorphine subcutaneous injection for treating PD can be traced back to 1951 when the drug was shown to have a major potential for relief of motor symptoms in PD.^42^ Therapy with the drug evolved over the next three decades in Europe, and some other countries with the discovery that domperidone can overcome nausea associated with apomorphine. In the late 1980s, open-label trials established the efficacy of apomorphine injection and infusion (Figure 4) in overcoming refractory “off” periods as well as attenuation of dyskinesia in PD.^43^ There is now Level 1 evidence from randomized, placebo-controlled studies available for apomorphine injection formulation, and a large-scale international placebo-controlled study is under way to evaluate the efficacy of apomorphine infusion versus placebo.^44,45^ Non-motor effects of apomorphine have also been researched and beneficial effects on sleep, mood, urinary function, and “off” related pain have been described.^46^ Injection is indicated for the rapid management of predictable “off” periods in PD such as early-morning “off” periods, whereas infusion is more appropriate for patients with multiple “off” periods, or refractory “off” periods. Skin nodules may complicate therapy as well as long-term issues with somnolence or other side effects associated with dopamine agonists.^47^

### Inhaled apomorphine (VR040)

The pulmonary system has a rich blood supply through its large surface area covered with capillaries. VR040 is a dry powder apomorphine formulation, which utilizes this route and is contained in a unit-dose blister. It aims to alleviate symptoms of “off” states in PD quickly and to provide an alternative application route to overcome problems with self-injecting and oral administration. In a single-center, phase 2, double-blind, placebo-controlled study patients received VR040 at three different doses (0.2, 0.5, and 0.8 mg). Patients with doses 0.5 and 0.8 mg achieved the on state, with the mean duration 40 and 20 min, respectively. They concluded that VR040 was rapidly absorbed, with peak concentration at 1–3 min and is well tolerated.^48^ Following promising results with the higher dose of 0.8 mg, another phase 2 study was conducted where 32 patients were given VR040 at four higher doses (1.5, 2.3, 3.0, and 4.0 mg). Analysis of pharmacokinetics revealed rapid establishment of peak plasma concentrations at 2–7 min post inhalation of VR040, translating into therapeutic reversal of “off” states within 10 min on average after dose. Safety of VR040 analysis showed no serious side effects, and fundamentally no clinically significant changes in electrocardiogram (ECG) or lung function were reported.^49^ Currently, VR040 is available for licensing in the United Kingdom, and is awaiting further studies to understand its efficacy, safety, and tolerability.^50^

### Apomorphine patch pump (ND0701)

This apomorphine-based product ND0701, is being developed to offer an alternative option to the currently available continuously administered apomorphine infusion for patients with severe PD. This product will be developed to be delivered by the pump patch technology and is included in the Neuroderm program to be used for advanced PD. This product will be particularly aimed at patients who may not respond to the levodopa patch–pump technology.^51^

### Sublingual apomorphine (APL-130277)

Apomorphine is commonly used for controlling the “off” periods in PD patients. APL-130277 is a thin-film strip in sublingual form, whereby absorption through the oral cavity mucosa allows for rapid delivery. Phase 2 trials have shown 15 of 19 patients turned fully “on” within 30 min (*P*<0.05) with a mean duration of 50 min.^52^ APL-130277 dosage can range from 10 to 30 mg; the mean effective dose is 18.4 mg.^53^ A pharmacokinetic study shows the blood concentration of sublingual and subcutaneous to be similar; therefore, a sublingual delivery option may allow for better patient compliance.^54^ Safety evaluation in the same participants showed APL-130277 to be generally well tolerated, with common dopaminergic medication side effects of dizziness (37%), somnolence (32%), and nausea (21%).^55^ Only one patient experienced orthostatic hypotension and no adverse events of dyskinesia or mucosal irritation was reported. APL-130277 has been shown to be effective in rapid conversion to “on” state irrespective of a patients' PD severity as defined by Hoehn and Yahr staging.^56^ It seems APL-130277 works faster than the subcutaneous form, giving real potential to its clinical use. This success from phase 2 trials has driven APL-130277 into two phase 3 trials.

### Buccal Zydis selegiline (ZELAPAR)

Selegiline hydrochloride is a selective and irreversible inhibitor of monoamine oxidase Type B, hence enabling a longer period of synaptic dopamine.^57^ ZELAPAR is an adjunct therapy to levodopa^58^ and uses selegiline hydrochloride in a freeze-dried orally disintegrating tablet, allowing for buccal mucosa absorption.^59^ Controlled trials have shown there to be faster absorption of ZELAPAR than conventional selegiline administration; however, plasma concentrations were significantly lower following 10 mg of ZELAPAR in comparison with conventional tablets.^60^ In another study, most significant reduction in “off” state time was observed at 2.5 mg dose.^61^ In a phase 4 trial, ZELAPAR was well tolerated and was preferred by patients because of the ease of use. However, it had no clinically significant change in efficacy over oral selegiline, which is cheaply available.^62^ Fowler *et al*.^63^ reported no significant difference between the form of selegiline administration (Zydis or transdermal), as well as all doses (2.5, 5.0, and 10 mg) well tolerated.^63^ There are currently no ongoing trials looking at ZELAPAR compared with other adjunct therapies.

### Subcutaneous rotigotine polyoxazoline

Polyoxazolines (POZs) are biodegradable bioconjugate polymers with potential in drug delivery.^64^ POZ–RTG conjugate, delivered subcutaneously, aims to provide continuous dopaminergic stimulation with greater control on drug loading and rate of release.^65^ *In vivo* studies, using 6-hydroxydopamine (6-OHDA) lesioned rat models, showed SER-214 (slow-release conjugate) to have a prolonged RTG half-life with reduced motor complications, which was sustained over repeated dosing.^66^ With these promising results, SER-214 has now Food and Drug Administration's approval to enter phase 1 study (NCT02579473) using *de novo* PD patients.^67^

### Other published treatment strategies

Furthermore, there are other treatment strategies that have been investigated as non-oral therapies for PD. These include the intranasal RTG and APO-MTD (Table 2). However, there are no ongoing or further studies on these drugs to date.

The liquid intranasal RTG is formulated of a pharmaceutically satisfactory acid addition salt of RTG and α-cyclodextrin. The α-cyclodextrin is used to predominantly stabilize the RTG hydrochloride used.^68^ A formulation for intranasal use of RTG has been developed for therapy in PD and restless leg syndrome. The formulation underwent two phase 2 studies to assess efficacy, safety, and tolerability in a randomized, double-blind, placebo-controlled, proof-of-concept manner. However, results of the studies did not show improvement in secondary outcome measures such as change in UPDRS III post administration and “off” reversals.^68^ The development of the drug was discontinued.

Priano *et al.*^69^ completed a pilot study on a new preparation of apomorphine, which was included in micromulsion and administration via the transdermal route (APO-MTD). Twenty-one patients were treated and the results obtained showed that APO-MTD delivered an average of 5.1 h of therapeutic plasma levels, improving the UPDRS III scores and reduced overall length of “off” periods. However, as promising as this treatment may seem, because of the time taken of 1 h to reach therapeutic concentrations, APO-MTD may not be the “ideal” treatment for the rapid relief of the “off” periods suffered by PD patients.^69^

The sublingual formulation of the D_2_–D_3_ agonist piribedil, S90049, was designed to abort “off” episodes in PD. A phase 2, double-blind, randomized, placebo-controlled study showed superiority of S90049 in UPDRS III post application in advanced-stage PD patients. In addition, the switch from “off” to “on” was significantly greater in patients using S90049 inhalation than placebo.^70^ Despite these results, no further activity has been reported since 2010.

## Conclusions

**Figure 5:**
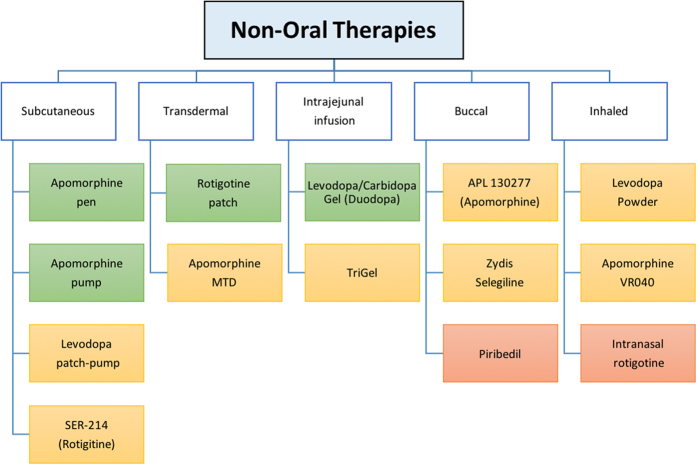
Diagram representing the non-oral therapies for Parkinson’s disease for different routes. The therapies are organized in terms of availability: clinically available (green), in development (yellow), and discontinued (orange). SER-214, rotigitine polyoxazoline conjugate; MTD, transdermal route; TriGel, levodopa carbidopa entacapone (liquid form); APL-130277, sublingual apomorphine.

In the last 5 years, existing knowledge about gastrointestinal dysfunction in PD with functional consequences on the oral drug-delivery strategy in PD have been extensively researched (Figure 5). Problems such as SIBO, altered intestinal microbiota, and delayed gastric emptying have added to the difficulties of oral therapy in PD, often culminating in commonly observed motor fluctuations such as delayed “on” or early-morning “off” sometimes associated with severe non-motor symptoms as well. Dysphagia, an often underestimated problem in PD, has been linked to silent aspiration with continued oral therapy, with silent aspiration being a risk factor for pneumonia. Existing non-oral therapies have served an important unmet need in PD and now have been shown to have not just motor but also significant non-motor beneficial effects and possibly with lower rates of ICD. This review highlights some significant short and long acting non-oral therapies in development or soon to be licensed, which will enhance the armamentarium of our treatment strategies for early and advanced therapy.

## Figures and Tables

**Table 1:** Existing and “in development” levodopa-based treatment strategies

*Route*	*Agent*	*Clinical positioning*
Subcutaneous	Levodopa belt pump	In development (phase 2 CT)
Subcutaneous/transdermal	Levodopa patch–pump	In development (phase 2 CT)
Intrajejunal infusion	Levodopa-carbidopa gel	In clinical use
	TriGel	In development (phase 1 CT)
Inhaled	Levodopa powder (CVT-301)	In development (phase 3 CT)

Abbreviation: CT, Clinical trial; TriGel, levodopa carbidopa entacopone (liquid form).

**Table 2:** Non-levodopa-based treatment strategies

*Route*	*Agents*	*Clinical positioning*
Subcutaneous	Apomorphine infusion (pump)	In clinical use
	Apomorphine injection (pen)	In clinical use
	Apormorphine patch–pump (ND0701)	In use, but not widely?
	Rotigotine polyoxazoline conjugate (SER-214)	In development (phase 1) and clinical studies on rat models published
Transdermal (patch)	Rotigotine	In clinical use as monotherapy and combined therapy
	Apomorphine (APO-MTD)	One clinical study with positive results in clinical motor efficacy and long action but no further studies
Buccal/sublingual	Apomorphine (APL-130277)	In development (phase 3)
	Zydis Selegiline	In development (ongoing phase 4)
	Piribedil	Halted development
Inhaled	Apomorphine (VR040)	In development (post-phase 2 clinical trial)
	Intranasal rotigotine	Completion of phase 2 but no further trials or studies/discontinued
